# Contact Adaption During Epidemics: A Multilayer Network Formulation Approach

**DOI:** 10.1109/TNSE.2017.2770091

**Published:** 2017-11-02

**Authors:** Faryad Darabi Sahneh, Aram Vajdi, Joshua Melander, Caterina M. Scoglio

**Affiliations:** Department of Electrical and Computer EngineeringKansas State University 5308 ManhattanKS66506

**Keywords:** Epidemics, contact adaptation, state-dependent switching networks, multilayer networks, nonlinear Perron-Frobenius

## Abstract

People change their physical contacts as a preventive response to infectious disease propagations. Yet, only a few mathematical models consider the coupled dynamics of the disease propagation and the contact adaptation process. This paper presents a model where each agent has a default contact neighborhood set, and switches to a different contact set once she becomes alert about infection among her default contacts. Since each agent can adopt either of two possible neighborhood sets, the overall contact network switches among $2^{N}$ possible configurations. Notably, a two-layer network representation can fully model the underlying adaptive, state-dependent contact network. Contact adaptation influences the size of the disease prevalence and the epidemic threshold—a characteristic measure of a contact network robustness against epidemics—in a nonlinear fashion. Particularly, the epidemic threshold for the presented adaptive contact network belongs to the solution of a nonlinear Perron-Frobenius (NPF) problem, which does not depend on the contact adaptation rate monotonically. Furthermore, the network adaptation model predicts a counter-intuitive scenario where adaptively changing contacts may adversely lead to lower network robustness against epidemic spreading if the contact adaptation is not fast enough. An original result for a class of NPF problems facilitate the analytical developments in this paper.

## 1 Introduction

Mathematical models of infectious diseases transmission are one of the primary tools for understanding the propagation of infectious diseases among plant, animal, or human populations [1], [2], [3]. Understanding how spreading dynamics are affected by individual-level transmission characteristics and large-scale properties of interactions aids endeavors to control and mitigate epidemics, making it critical for the public health and security.

In addition to their critical role in public health decision making [4], infectious disease models are appealing from complex systems perspective. Take for instance the Susceptible-Infected-Susceptible (SIS) model [3], where each individual in the population is either ‘*Susceptible*’ or ‘*Infected*’. The SIS model simply states that susceptible individuals may become infected when interacting with infected individuals, and infected individuals will become susceptible immediately after recovery. Rich dynamics of the SIS model, such as the phase transition observed between fast die-out of infections and long-term epidemic persistence [5], exemplify the ability of simple individual-level interactions to give rise to emergent phenomena.

Understanding disease transmission dynamics in human social networks is particularly challenging [6], partly because humans take preventive measures and alter their interactions in response to disease spreading [7], which subsequently change the course of the spreading [8]. As such, coupled modeling of behavioral change and infection transmission dynamics has seen significant attention recently [8], [9], [10], [11]. Medical treatments, quarantines, illness management practices, and individual preventive behaviors are a few examples of ways society works to reduce disease spreading.

Common preventive behaviors of individuals to the emergence of an epidemic are (1) adopting hygiene/pharmaceutical actions such as wearing a mask, using condoms, improving bodily/environmental cleanliness, and receiving vaccinations, and (2) altering contacts to avoid infection. In the first case, individuals are intending to reduce the probability of infection by cleansing themselves and their environment—or at least placing barriers between the two [12], [13], [14], [15], [16], [17]. In the second case, when individuals change who they come in contact with, the fundamental topology of the network itself is changing. As individuals remove certain contacts with people, while possibly creating new ones, the structural paths available to dynamic processes are being altered, resulting in rich dynamic interplay between network topology and the spreading process on top of it [18], [19], [20], [21], [22], [23], [24], [25], [26].

Existing approaches to incorporate preventive behaviors in mathematical models of infectious diseases fall into two general categories. First approach incorporates the effect of preventive behaviors directly into disease model parameters [27], [28], [29], [30], [31], [32], [33]. The second approach introduces additional dynamic states into a disease model to explicitly distinguish those who have adopted a preventive behavior from those who have not [13], [17], [34], [35], [36], [37], [38]. One example of individual-based models taking the second approach is the susceptible–alert–infected–susceptible (SAIS) framework, first introduced in [17].

The SAIS framework adds an ‘*Alert*’ state to the networked SIS model of [39]. The alert state represents individuals who (similar to susceptible individuals) can potentially become infected, but has adopted a preventive behavior. In the original SAIS model [17], alert individuals have a lower infection rate compared to the susceptible individuals, and susceptible individuals could become alert in presence of infection among their local contacts. The lower infection rate of alert individuals would correspond to their type-1 preventive behaviors (such as wearing masks or using condoms). This model predicts possibility of total eradication of an epidemics through preventive behaviors[34]. In a subsequent study [40], authors considered an information-dissemination network as an alternative alerting mechanism, and proposed the optimal design solution for an information-dissemination network based on eigenvector centralities [41] in the contact network graph. The SAIS model has been further explored in [42], [43], [44].

In this paper, we introduce the AC-SAIS model, where AC stands for ‘**A**daptive **C**ontact’, to model a scenario in which individuals change their contact neighborhood upon becoming alert. More specifically, each susceptible individual $i$ is in contact with a given set of individuals $(\mathcal{N}^S_i)$, and when she becomes alert, she switches to another set of individuals $(\mathcal{N}^A_i)$. We will use the terms *default neighborhood* and *adapted neighborhood* to distinguish the two. In our model, we assume both of these neighborhoods are known *a priori*. Yet, we do no make any restrictive assumptions on these neighborhood sets and deliver our results in the most generic setup. In practice, the default and adapted neighborhood sets might be closely related. For example, in a social distancing scenario [45], the adapted neighborhood would be a subset of the default neighborhood. Social distancing is not the only possible scenario of contact adaptation. In the context of sexually transmitted infections, for example, when a person is notified that one of his sexual partners is infected, in response, he may abandon all or some of his set of partners and seek partnership from a new venue.

When nodes adapt their contacts to a neighborhood constituting a more robust network, one might intuitively expect that the robustness of the network against epidemic spreading increases monotonically with the contact adaptation rate. This is true in the case of social distancing (where the alert neighborhood is a subset of the default ones) as it always help mitigating epidemic spreading, and the faster the social distancing is implemented, the better. However, when the set of adapted contacts of an individual is not restricted to be a subset of their default contacts, the network robustness against epidemic spreading can be a non-monotone function of the contact adaptation rate. Indeed, our model detects a counter-intuitive scenario where adaptively changing contacts may adversely lead to lower network robustness against epidemic spreading if the adaptation is not fast enough.

From dynamical systems perspective, this study contains several contributions. First, we propose a novel state-dependent switching network framework and show that a multilayer-network [46] formulation can be successfully employed. Second, we develop an original result of nonlinear Perron-Frobenius theory, where we find necessary and sufficient conditions for existence and uniqueness of a strictly positive eigenvector for the class of non-negative, concave maps. We apply this tool to find the epidemic threshold for our AC-SAIS model. Furthermore, we introduce a novel notion of connectivity for multilayer networks, which is novel for the new research field of multilayer networks.

The rest of the paper is organized as follows: After the literature review in Section 2, Section 3 introduces a novel notion of multilayer connectivity and an original result for nonlinear Perron-Frobenius theory, which are pivotal for the subsequent modeling and analysis. Section 4 develops the AC-SAIS model, showing that the proposed adaptive contact can be equivalently modeled by multilayer networks. Analyses in Section 5 are followed by numerical experiments in Section 6. Several proofs to theorems and lemmas are omitted for the sake of brevity, and can be found in the Supplemental Materials of this article, which can be found on the Computer Society Digital Library at http://doi.ieeecomputersociety.org/10.1109/TNSE.2017.2770091.

## 2 Literature Review

Typical approaches to modeling spreading processes on networks consider network topologies as independent of individual node states, such is the case when nodes retain the same set of contacts regardless of whether or not they, or their neighbors, are infected. This assumption is made for simplicity’s sake and is not representative of real world networks; especially in regards to social networks where a person’s contacts are in constant fluctuation. The notion of state-dependent topologies is especially poignant in the context of disease dynamics where a person will adjust who they come in contact with when in the presence of an infection. The extent to which this occurs can vary greatly—from removing a single contact to completely changing all of them—depending on the perceived severity of an infection.

Several formulations of adaptive contact exist in the literature of infectious disease modeling, including: 1) social distancing [47], where healthy individuals lower their contact with the rest of the population, 2) delete-and-reactivate [48], where healthy break their contact with infected population and reactivate after some time, 3) rewiring [49], [50], where healthy break their contact with infected population and create new links with healthy members [21] or any other randomly chosen individual [23].

Altering the local contacts can have a strong effect on disease dynamics, which in turn influences the contact adaptation process; a complicated mutual interaction between a time varying network topology and the dynamics of the nodes emerges. For example, Gross et al. [21] presented a model where susceptible individuals rewire their links from other infected individuals toward susceptible ones in an SIS model, resulting in the formation of two loosely connected clusters. Several researches have built on this model: Marceau et al. [22] additionally include the infection state of its neighbors in the node information. Risau et al. [23] rewire susceptible individuals from infected neighbors to random nodes, which in some cases completely suppresses epidemic spreading.

Most of contact adaptation schemes have been implemented for well-mixed populations or random network models of physical interactions. Studies that work with generic graphs as their contact network are scarce in the literature. Among a few existing research endeavors is the Adaptive-SIS (ASIS) model developed by Guo et al. [48], who studied an SIS epidemic model where contacts between susceptible and infected nodes are removed at some rate and reactivated later. They showed the epidemic threshold increases as a function of the link removal rate, while the network topology exhibits binomial-like degree distribution, assortative mixing, and modularity. This approach was rigorously extended by Ogura and Preciado [51], who additionally considered heterogeneous node and edge parameters, as well as a method for optimizing adaptation rates to mitigate epidemic outbreaks. This approach of adaptation for generic graphs considers a dynamic equation for the edge weights which is coupled with the epidemic model. Another approach would be through the notion of switching networks in dynamical systems.

A switching contact network is defined as a set of distinct networks where the “active” network at any given time is determined by some switching signal. More precisely, we denote a switching network $G(t)=(V,E^{s(t)})$, where $s(t):\mathbb {R}\rightarrow \lbrace 1,2,\ldots, q\rbrace$ is a signal that determines which of the $q$ networks are active at time $t$. Usually this signal is external and independent of the system states. For example, a common approach is to consider $s(t)$ as a Markov process independent from the disease states [52], [53]. The collection of possible edge sets $\mathcal{E}=\lbrace E^1,E^2,\ldots, E^q\rbrace$ may be given *a priori* as in [52], or they might be generated from local processes as in [53]. In the latter, Ogura and Preciado considered a base graph with $|E|$ edges where each edge can become active or inactive according to an externally defined Markov process, leading to an overall $2^{|E|}$ possible configurations for the switching contact network. We can also think of a more complex situation where the switching signal is dependent on the system states. In this way, the topology of the active network determines the evolution of the dynamic process and in turn, the state of the process itself signals network switching. Here lies our proposed contact adaptation scheme.

We consider a class of switching networks where the neighborhood set of each node depends on the state it occupies. Specifically, each node $i$ has one of two contact sets $\mathcal{N}^S_i$ and $\mathcal{N}^A_i$, depending on whether is is ‘susceptible’ or ‘alert’. Therefore, for a network of size $N$, the entirety of the switching network is composed of $2^{N}$ separate topologies. In this case, not only the network state-space size exponentially increases by $N$, but also the switching signal depends on the collective system state.

## 3 Fundamental Concepts and Tools

Before diving into the modeling and analysis, we first start with a novel notion of connectivity for multilayer networks and an original results for a class of nonlinear Perron–Frobenius problems that will facilitate the subsequent developments in this paper.

### 3.1 Nonlinear Perron Frobenius

The classical Perron-Frobenius theorem [54] concerns the eigenvalue problem $Ax=\lambda x$ for a nonnegative and irreducible matrix $A$. Let $\mathbb {R}_{+}^{n}$ be the non-negative cone in the $n-$dimensional Euclidean space

\begin{equation*}
\mathbb {R}_{+}^{n}=\lbrace x\in \mathbb {R}^{n}\vert x_{i}\geq 0 \quad \mathrm{for} \quad 1\leq i\leq n\rbrace .
\end{equation*}

Assuming $x,y \in \mathbb {R}^{n}_{+}$, here $x\preceq y$ ($x\prec y$) means $x_{i}\leq y_{i}$ ($x_{i}<y_{i}$) for $1\leq i\leq n$ and $x \;\precnsim\; y$ denotes $x\preceq y$ but $x\ne y$. A matrix $A=[a_{ij}]$ is called non-negative if all of its entries are either positive or zero. We can construct a graph $G(A)$ associated with $A$ such that the edge $(i,j)$ exists if $a_{ij}>0$. The matrix $A$ is irreducible if and only if its associated graph $G(A)$ is strongly connected. The classical Perron-Frobenius theorem may be stated as the following:

**Theorem 1 (Perron–Frobenius Theorem [54]).**

Let $A$ be a nonnegative, irreducible matrix. Then $A$ has a positive eigenvalue $\lambda _1>0$ which has multiplicity one and any eigenvalue of $A$ has a magnitude smaller than or equal to $\lambda _1$. Furthermore the eigenvector $\boldsymbol{v}_1$ corresponding to $\lambda _1$ is strictly positive (i.e., $\boldsymbol{v}_1\succ 0$) and is the only eigenvector of $A$ in the nonnegative cone.

From mappings perspective, the classical Perron–Frobenius theory concerns solutions to the eigenvalue problem $F(x)=\lambda x$ where $F(x)=Ax$ is a linear self-map of the non-negative cone. By “self-map of the non-negative cone,” we mean that $F:\mathbb {R}_{+}^{n}\rightarrow \mathbb {R}_{+}^{n}$ maps the non-negative cone to itself. But what if the map $F(x)$ is not linear? Can we still get powerful results for nonlinear maps analogous to the Perron–Frobenius theorem? The whole area of the nonlinear Perron–Frobenius theory [55], [56], [57], [58], [59] seeks answer to these questions. A thorough review of nonlinear Perron–Frobenius theory is out of the scope of this paper. In short, results are usually more limited in that existence, uniqueness, or strictly positivity of an eigenvector is seldom guaranteed unless under restrictive assumptions on the nonlinear map.

The following properties are among the possibilities to relax the linearity assumption for the non-negative map $F$. Note that the linear map $F(x)=Ax$ with non-negative matrix $A$ has all of these properties.

**Definition 1.**

Assume $F:\mathbb {R}_{+}^{n}\rightarrow \mathbb {R}_{+}^{n}$ is a self-map of nonnegative cone. We say $F$ is

1) *homogeneous*, if for any $x\succeq 0$ and $c\geq 0$, $F(cx)=cF(x)$,
2) *concave*, if $F(\theta x+(1-\theta)y)\succeq \theta F(x)+(1-\theta)F(y)$ for all $x,y\succeq 0$ and $0\leq \theta \leq 1$,
3) *super-additive*, if $F(x+y)\succeq F(x)+F(y)$ for all $x,y\succeq 0$,
4) *monotone,*^1^1.Sometimes, this property is referred to as *order-preserving*. if $F(y)\succeq F(x)$ for all $y\succeq x \succeq 0$.

The homogeneity property indicates that if $x^*\succeq 0$ is an eigenvector of $F$, so is $cx^*$ for any $c\geq 0$. Furthermore, the following lemma indicates that the class of homogeneous, concave self-maps of the non-negative cone is a special case of homogeneous, monotone maps.

**Lemma 1.**

If $F:\mathbb {R}_{+}^{n}\rightarrow \mathbb {R}_{+}^{n}$ is a homogeneous, concave map of the non-negative cone, then $F$ is also monotone and super-additive.

Several results in the literature concern the more general class of homogeneous, monotone maps [56], [57]. While existence and strict positivity of an eigenvector can be proved for this class of maps, uniqueness cannot be guaranteed without quite restrictive assumptions [56]. For example,^2^2.This example is from [56]. for the homogeneous, monotone function $F(x)=[\max \lbrace x_1,\frac{x_2}{2}\rbrace, \max \lbrace \frac{x_1}{2},x_2\rbrace ]^T$, any vector $[x_1,x_2]^T\in \mathbb {R}_+^2$ with $\frac{x_1}{2}\leq x_2 \leq 2x_1$ is an eigenvector of $F$ with eigenvalue $\lambda =1$. On the contrary, existence and strict positivity of a unique eigenvector can be proved for the special class of homogeneous, concave maps.

The nonlinear map of interest in this paper falls in the special class of homogeneous and concave maps. Therefore, we focus on this class of nonlinear maps and develop a new result.

So far, we relaxed the linearity restriction by assuming that our nonlinear map is homogeneous and concave. The next question is what would be the counter part to irreducibility of $A$ in the linear map $F(x)=Ax$ for a homogeneous, concave map. For homogeneous, concave maps, Krause [58] proposes the following condition:

**Definition 2 (Krause [58], Section 3).**

We say the homogeneous, concave self-map $F:\mathbb {R}_{+}^{n}\rightarrow \mathbb {R}_{+}^{n}$ satisfies condition^3^3.In Krause [58], authors refer to this condition as being *irreducible*. We choose to avoid this term to avoid any confusion with other notions that tend to extend irreducibility of linear maps to nonlinear domain.
**C1** in $\mathbb {R}_{+}^{n}$ if for any non-empty subset $\emptyset \ne J \;\subsetneq\; \lbrace 1,\ldots, n\rbrace$, there exists $j\in J$ and $i\notin J$ such that $F_i(e_j)>0$, where $e_j$ is the $j$th unit vector in $\mathbb {R}^n$ and $F_i$ denotes the $i$th component of $F$.

Furthermore, Krause proves that condition **C1** is a *sufficient condition* for existence and uniqueness of a positive eigenvector:

**Theorem 2 (Krause [58], Theorem 13).**

For the self-map $F:\mathbb {R}_{+}^{n}\rightarrow \mathbb {R}_{+}^{n}$, which is concave, homogeneous, and satisfies condition **C1**, the equation $F(x)=\lambda x$ has a strictly positive solution $x=x^*\succ 0$, $\lambda =\lambda ^*>0$, and $x^*$ is the only eigenvector in the non-negative cone (up to scaling).

We argue that the condition **C1** for the notion of irreducibility in [58] may be restrictive, and same strong results would be still valid under a more relaxed condition. Indeed, the nonlinear map of our interest in this paper may not satisfy the condition **C1** in Definition 2.

To illustrate, suppose $n=3$ and the nonlinear map is $F(x)=[\min \lbrace x_2,x_3\rbrace, x_1+x_3,x_1+x_2]^T$. This map is both homogeneous and concave. However, it does not satisfy condition **C1** of [58] stated in Definition 2. To test this, let $J=\lbrace 2,3\rbrace$; no $j\in J$ leads to $F_1(e_j)>0$ because $F(e_2)=e_3$ and $F(e_3)=e_2$. However, this map has a unique, strictly positive eigenvector $x^*=[\frac{1}{1+2\lambda ^*},\frac{\lambda ^*}{1+2\lambda ^*},\frac{\lambda ^*}{1+2\lambda ^*}]^T$ and $\lambda ^*=\frac{1+\sqrt{5}}{2}$ with $||x^*||_1=1$. Another example is $F(x)=[\frac{x_2x_3}{x_2+x_3},x_1+x_3,x_1+x_2]$. Again, $F(e_2)=e_3$ and $F(e_3)=e_2$, so it does not satisfy condition **C1**. However, this map has a unique, strictly positive eigenvector $x^*=[\frac{1}{1+4\lambda ^*},\frac{2\lambda ^*}{1+4\lambda ^*},\frac{2\lambda ^*}{1+4\lambda ^*}]^T$ and $\lambda ^*=\frac{1+\sqrt{3}}{2}$ with $||x^*||_1=1$.

**Definition 3.**

We say the homogeneous, concave self-map $F:\mathbb {R}_{+}^{n}\rightarrow \mathbb {R}_{+}^{n}$ of the non-negative cone satisfies condition **C2** in $\mathbb {R}_{+}^{n}$ if for any choice of $\emptyset \ne J \;\subsetneq \lbrace 1,\ldots, n\rbrace$, there exists $i\notin J$ such that $F_i(e_J)>0$, where $e_J$ is defined as $e_J\triangleq \sum _{j\in J}e_j$.

The example function $F(x)=[\min \lbrace x_2,x_3\rbrace, x_1+x_3,x_1+x_2]^T,$ which does not satisfy condition **C1**, does satisfy **C2**. For instance, selecting $J=\lbrace 2,3\rbrace$ yields $F_1(e_J)>0$ because $F(e_J=[0,1,1]^T)=[1,1,1]^T$. The following lemma proves that **C2** is indeed less restrictive than **C1**.

**Lemma 2.**

A homogeneous, concave self-map $F$ of the nonnegative cone that satisfies condition **C1** also satisfies condition **C2**.

We would like to emphasize that there is nothing special about usage of $e_J$ in Definition 3. The following lemma shows that any vector that has positive values on elements corresponding to $J$ and is zero on other elements would be equivalently applicable.

**Lemma 3.**

For any choice $x\succ 0$, we have $F_i(x\circ e_J)>0$ if and only if $F_i(e_J)>0;$ where the symbol $\circ$ denotes the Hadamard (entry-wise) multiplication.

In the linear domain, we know that if a non-negative matrix $A$ is irreducible, the matrix $A+cI$ is primitive for any $c>0$ [60, Theorem 9], and vice versa. How would be the extension of this idea to the nonlinear domain? First, let us precisely define a primitive map.

**Definition 4.**

The self-map $H:\mathbb {R}_{+}^{n}\rightarrow \mathbb {R}_{+}^{n}$ of the non-negative cone is called *primitive* if there exists $M$ such that $H^m(x)\succ 0$ for all $m\geq M$ and $x \;\succnsim\; 0$. Here, $H^{m}$ denotes the $m$th iterate of $H$, i.e., $H^m(x)=H(H^{m-1}(x))$ and $H^0(x)\triangleq\; x$.

The following theorem states that $F$ satisfying **C2** and $F(x)+cx$ being primitive are equivalent.

**Theorem 3.**

The map $F_c(x) \triangleq \;cx+F(x)$ with $c>0$ is primitive if and only if the homogeneous, concave self-map $F:\mathbb {R}_{+}^{n}\rightarrow \mathbb {R}_{+}^{n}$ of the non-negative cone satisfies condition **C2**.

The duality between $F$ satisfying **C2** and $F(x)+cx$ being primitive leads to the main theorem in this paper:

**Theorem 4.**

Statements of Theorem 2 still holds if condition **C1** is replaced with condition **C2**. Furthermore, if $x^*\succ 0$ is a unique eigenvector of the homogeneous concave map $F$ in $\mathbb {R}_+^N,$ then $F$ must satisfy condition **C2**. Moreover, iterations of $F_c(x)$ with $c>0$ converge to $x^*,$ i.e.,

\begin{equation*} \lim_{k\rightarrow \infty } \bar{F}_{c}^{k}(x)=x^*, \; \mathrm{ for \; all \; x \;\succnsim\; 0, \; and } \; \bar{F}_c(x)\triangleq \frac{F_c(x)}{||F_c(x)||}. \end{equation*}

Compared with Theorem 1, it is evident that results for the nonlinear Perron–Frobenius problem in case of homogeneous, concave maps are very strong; existence and uniqueness of a strictly positive eigenvector can be guaranteed. Our contribution to the theory of nonlinear Perron–Frobenius theory for homogeneous, concave maps is that we relaxed the sufficient condition of [58] (through replacing **C1** by **C2**) and proved that this new^4^4.We got the inspiration for our definition of condition **C2** for homogeneous, concave maps from a notion in [56] for homogeneous, monotone maps. Gaubert and Gunawardena [56] refer to the homogeneous, monotone map $F$ as *indecomposable* if for any choice of $\emptyset \ne J \;\subsetneq\; \lbrace 1,\ldots, n\rbrace$, there exists $i\notin J$ such that $\lim _{a\rightarrow \infty }F_i(r_J(a))=\infty$, where $r_J(a)$ is defined as $(r_J(a))_j=a$ if $j\in J,$ and $(r_J(a))_j=1$ otherwise. While this may look very similar (or perhaps equivalent) to condition **C2**, we would like to point out a subtle difference which can be very consequential. Consider the function $F(x)=[\frac{4x_1^{\frac{1}{2}}x_2^{\frac{3}{2}}}{x_1+x_2}, x_1+x_2]^T$. This function is homogeneous, concave, and monotone. It does not satisfy condition **C2** because for $J=\lbrace 2\rbrace$ we get $F_1(e_J)=F_1(e_2)=0$. However, it falls in the category of indecomposable maps of [56] because $\lim _{a\rightarrow \infty }F_1(r_2(a))=\lim _{a\rightarrow \infty }\frac{4a^{\frac{3}{2}}}{1+a}=\infty$ and $\lim _{a\rightarrow \infty }F_2(r_1(a))=\lim _{a\rightarrow \infty }1+a=\infty$. The nonlinear eigenvalue problem for this function gives two eigenvectors in $\mathcal{R}_+^2$, namely, $x_1^*=[1,1]^T$ with $\lambda _1=2$, and $x_2^*=[0,1]^T$ with $\lambda _2=1$; which is consistent with the fact that it does not satisfy condition **C2**. condition is also the necessary condition for uniqueness of the eigenvector in the non-negative cone.

### 3.2 Multilayer Networks and an Algorithmic Notion of Connectivity

Graph theory is the mathematics of networks. In graph theory, a directed graph is formally defined as an ordered pair $G=(V,E)$, where $V$ is the set of nodes and $E\subset V\times V$ is the set of ordered pairs of nodes representing their directed relation. We say node $j$ is a neighbor of node $i$, if $(i,j)\in E$. The set $\mathcal{N}_i=\lbrace j|(i,j)\in E\rbrace$ denotes the neighbors of node $i$. A path $(v_0=i,v_1,\ldots, v_{l-1},v_l=j)$ of length $l$ is an ordered tuple of edges than connects $i$ to $j$, i.e., $(v_{k-1},v_k)\in E$. A directed graph is strongly connected if there exists a path between all ordered pair of nodes in the network [54].

Several natural and technological systems show complex patterns of interactions among their heterogeneous entities. To capture the complexities of such systems, the network science community has recently shown substantial interest in the notion of multilayer networks [46], [61] and developing proper mathematics for them beyond the classical graph theory [62].

In this paper, we denote a *multilayer* network^5^5.In some literature, this may be referred to as a *multiplex* network. as an ordered tuple $\mathcal{G}=(V,E_A,E_B)$ where nodes in $V$ are connected through two link types $E_A$ and $E_B$. Corresponding to the multilayer network $\mathcal{G}$, we define $G_A=(V,E_A)$ and $G_B=(V,E_B)$ as the *layers* of $\mathcal{G}$. Motivated by the notion of strong connectivity for directed graphs, we propose a novel notion of connectivity for multilayer networks in the following.

Our proposed notion of multilayer connectivity, which from now on we will refer to it as *M–connectivity*, has an algorithmic definition. To motivate and acquaint our definition to the reader, we first point out a straight-forward property of simple strongly connected graphs. Suppose for the graph $G=(V,E)$ we have an arbitrary partition $\mathcal{P}$ of the node set $V$, i.e., members of $\mathcal{P}$ are non-empty disjoint subsets of $V$ that cover $V$, more precisely

\begin{align*}
&(1)\ 
\emptyset \notin \mathcal{P},\\
&(2)\ 
I\cap J=\emptyset \; \mathrm{ for \; any } \; I\ne J\in \mathcal{P}\\
&(3)\mathop{\bigcup} _{I\in \mathcal{P}}I=V.
\end{align*}

We can build a graph $\boldsymbol{G}=(\mathcal{P},\mathcal{L})$, where the partition set $\mathcal{P}$ is the node set of $\boldsymbol{G}$. Note that each node $I\in \mathcal{P}$ of $\boldsymbol{G}$ is a partitioning subset of $V$. As such, to avoid possible confusion, we will refer to nodes of $\boldsymbol{G}$ as *hypernodes* from now on. We assign a directed link from one hypernode $I\in \mathcal{P}$ to another hypernode $J\in \mathcal{P}$, if there is a node $i\in I$ of $G$ that is connected to a node $j\in J$, i.e., $(i,j)\in E$. Trivially, yet importantly, strong connectivity of $G$ implies strong connectivity of $\boldsymbol{G}$. For a multilayer network $\mathcal{G}$, we use a related notion to define connectivity.^6^6.We have been inspired by the notions of indecomposability for nonlinear maps and the method of aggregated graphs from Gaubert and Gunawardena [56, Sections 1.3 & 3.4]. The main difference is that connection among subsets must be through both layers. Following provides a formal definition.

For a multilayer network $\mathcal{G}=(V,E_A,E_B)$, we iteratively build graphs $\boldsymbol{G}^k=(\mathcal{P}_k,\mathcal{L}_k)$, starting with $\boldsymbol{G}^0=(\mathcal{P}_0,\emptyset)$, where $\mathcal{P}_0=\lbrace \lbrace 1\rbrace, \lbrace 2\rbrace, \ldots, \lbrace N\rbrace \rbrace$ is the trivial partition of $V$ singletons. From the graph $\boldsymbol{G}^{k-1}$, we build $\boldsymbol{G}^k=(\mathcal{P}_k,\mathcal{L}_k)$ in the following way:

*Step 1.* Define the hypernode set $\mathcal{P}_k$ of cardinality equal to the number of strongly connected components of $\boldsymbol{G}^{k-1}$ where each element $I\in \mathcal{P}_k$ groups one and only one strongly connected component of $\boldsymbol{G}^{k-1}$ (i.e., $I$ is the union of all the hypernodes in that strongly connected component). Note that, doing so, the hypernode set $\mathcal{P}_k$ always denotes a partitioning of the node set $V$.

*Step2.* We assign the directed link $(I,J)\in \mathcal{L}_k$ if at least one *single* node in $I$ is connected to $J$ through both layers *simultaneously*,^7^7.Note that this is different from, $\exists i_1,i_2\in I~~\mathrm{s.t.}~~(i_1,j_{1})\in E_A,\ 
(i_2,j_{2})\in E_B~~\mathrm{for \; some}~~j_{1},j_{2}\in J$, which basically indicates that both individual layers $G_A$ and $G_B$ are strongly connected. i.e.,

\begin{align*}
\mathcal{L}_k=\bigg \lbrace (I,J)\in & \;\mathcal{P}_k\times \mathcal{P}_k\Big \vert \exists i\in I \quad \mathrm{s.t.} \quad (i,j_{1})\in E_A,\\
& \qquad (i,j_{2})\in E_B \quad \mathrm{for \; some} \; j_{1},j_{2}\in J\bigg \rbrace .
\end{align*}

**Fig. 1.**
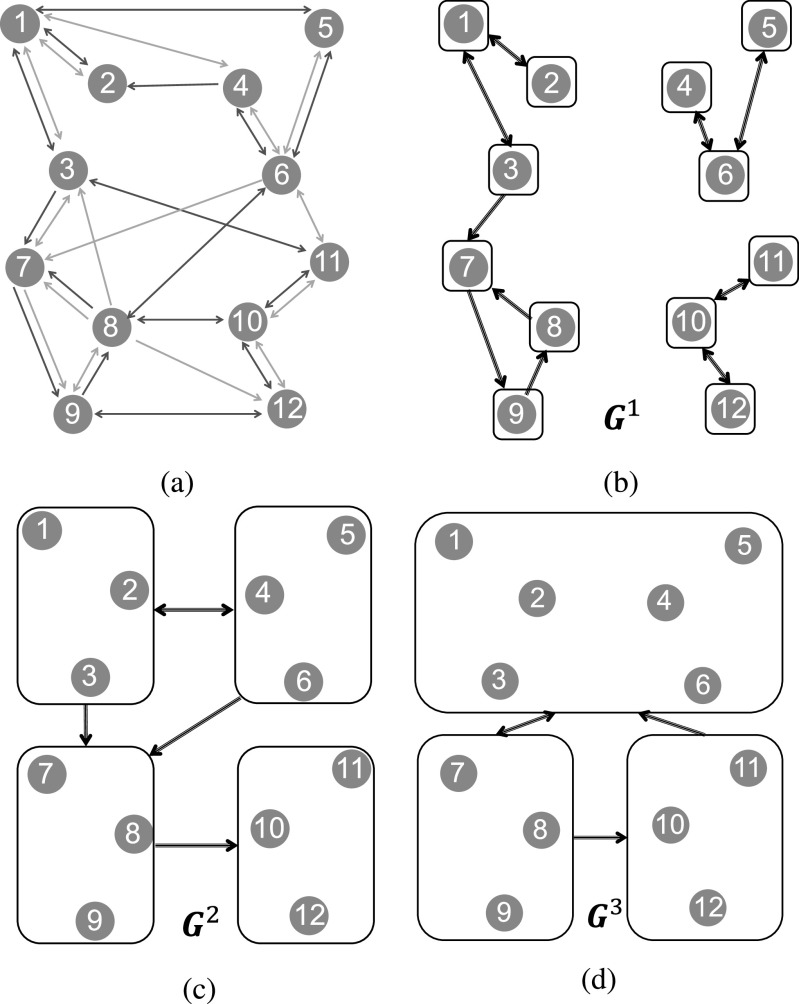
Example of an M-connected multilayer network according to Definition 1. (a) In the two-layer graph, the red arrows represent $E_A$ edges and green arrows represent $E_B$ edges. (b) The first graph $\boldsymbol{G}^1$ has the hypernodes set $\mathcal{P}_1=\lbrace \lbrace 1\rbrace, \lbrace 2\rbrace, \ldots, \lbrace 12\rbrace \rbrace$, and its links are the intersection of $E_A$ and $E_B$ edges. Hypernodes are depicted by black squares, and links between them are shown by black arrows. The graph $\boldsymbol{G}^1$ is not strongly connected. (c) The second aggregate graph $\boldsymbol{G}^2$ has the strongly connected components of $\boldsymbol{G}^1$ as its hypernodes set $\mathcal{P}_2=\lbrace \lbrace 1, 2, 3\rbrace, \lbrace 4,5,6\rbrace, \lbrace 7,8,9\rbrace, \lbrace 10,11,12\rbrace \rbrace$. The links among the hypernodes is according to Step 2 in Section 3.2. For example, the directed link $(\lbrace 4,5,6\rbrace, \lbrace 7,8,9\rbrace)\in \mathcal{L}_2$ is due to node 6, a member of $\lbrace 4,5,6\rbrace$, being connected to the hypernode $\lbrace 7,8,9\rbrace$ through both layers (because, $(6,8)\in E_A$ and $(6,7)\in E_B.$). The graph $\boldsymbol{G}^2$ is not strongly connected either. (d) The third aggregated graph $\boldsymbol{G}^3$ groups strongly connected components of $\boldsymbol{G}^2$ as its hypernodes set $\mathcal{P}_3=\lbrace \lbrace 1,2,3,4,5,6\rbrace, \lbrace 7,8,9\rbrace, \lbrace 10,11,12\rbrace \rbrace$. The graph $\boldsymbol{G}^3$ is strongly connected. Therefore, the two-layer network in (a) is M–connected according to Definition 1.

Fig. 1 illustrates the iterative procedure explained above.

**Definition 5.**

A multilayer network $\mathcal{G}=(V,E_A,E_B)$ is *M– connected*, if starting with with $\boldsymbol{G}^0=(\mathcal{P}_0,\emptyset)$—where $\mathcal{P}_0=\lbrace \lbrace 1\rbrace, \lbrace 2\rbrace, \ldots, \lbrace N\rbrace \rbrace$ is the trivial partition of $V$ singletons—and inductively building $\boldsymbol{G}^1,\boldsymbol{G}^2,\ldots$ following Step 1 and Step 2 described above, there exists an iteration step $k_*$ such that $\boldsymbol{G}^{k_*}$ is strongly connected.

Intuitively, M–connectivity of $\mathcal{G}$ implies that if we split the node set $V$ into any two subsets $V_a$ and $V_b$, there is always a node in $V_a$ (resp. $V_b$) that is connected to $V_b$ (resp. $V_a$) through both edge types. A necessary condition for M–connectivity of $\mathcal{G}$ is that both individual layers $G_A$ and $G_B$ are strongly connected. Moreover, a sufficient condition for M–connectivity of $\mathcal{G}$ is that the intersection graph $G_c\triangleq (V,E_A\cap E_B)$ is strongly connected (because, $\boldsymbol{G}^1$ which is similar to $G_c$, will be already strongly connected).

*M–Connectivity and Condition C2.* Consider a multilayer network $\mathcal{G}=(V,E_A,E_B)$, where the node set are labeled from 1 to $N$, i.e., $V=\lbrace 1,2,\ldots, N\rbrace$. By defining a real valued vector $x:V\rightarrow \mathbb {R}_+^N$ on node set $V$, we show the relation between M–connectivity and condition **C2** for functions $F(x)$.

**Theorem 5.**

Associated with the multilayer network $\mathcal{G}=(V,E_A,E_B),$ where $V=\lbrace 1,2,\ldots, N\rbrace$, suppose a homogeneous, concave map $F$ of the non-negative cone is such that for any nontrivial subset $J$ of $V$ and $i\notin J$, $F_i(e_J)>0$ if and only if there exists $j_1,j_2\in J$ for which $(i,j_1)\in E_A$ and $(i,j_2)\in E_B$. Then, $F$ satisfies condition **C2** if and only if the multilayer network $\mathcal{G}$ is M-connected.

## 4 Model Development

Before introducing our model, we first review a background on the networked SIS epidemic process.

### 4.1 A Background on Networked SIS Model

Susceptible–infected–susceptible model is a paradigmatic epidemic spreading model. In the SIS model, each individual is either *susceptible* or *infected*, and individuals are assumed to immediately become susceptible to the disease after recovery. SIS model is thus suitable for modeling sexually transmitted infections such as Gonorrhea and Syphilis [2].

Classical compartmental epidemic models assume homogeneous (fully mixed) interactions among individuals. In networked epidemic models, interactions among individuals are explicitly modeled using a *contact network*, represented by the graph $G=(V,E)$, where individuals are represented by nodes $V$ of a graph and possible interactions are the edges $E$ of a graph. Node $j$ is a neighbor of node $i$, denoted as $(i,j)\in E$, if she can infect node $i$ directly. We can also use weighted graphs to represent contact networks. Doing so, the weight value of a link would serve as a proxy for heterogeneity of contact levels among pairs of individuals. For example, if both nodes $j$ and $k$ are infected and $w_{ij}=2w_{ik}$, the likelihood that a susceptible node $i$ contracts the disease from node $j$ is double the likelihood of contracting it from node $k$. In this paper, we allow the contact graph be directed and weighted.

In the networked SIS model [63], the state of node $i$ at time $t$ is denoted by $X_{i}(t)\in \lbrace S,I\rbrace$, where $X_{i}(t)=S$ if the node is susceptible or $X_{i}(t)=I$ if it is infected. In this model, a susceptible nodes becomes infected if it is exposed to an infected individual. Moreover, an infected individual recovers and becomes susceptible again after a recovery period. The infection and curing times are commonly assumed to have a memoryless property, leading to exponentially distributed time intervals in continuous time descriptions. More general time distributions are also possible and addressed in the literature to some extent [53], [64], [65], [66], [67].

The overall evolution of the nodes states are due to their interactions with each other. Hence, mathematical description of the SIS model requires utilization of the collective state $\boldsymbol{X}=[X_{1},\ldots, X_{N}]$, which is the joint state of all $N$ nodes in the network. The network state is a continuous-time Markov process that undergoes transition over a space consisting of $2^{N}$ possible network states. In this description, we say an event has occurred if the state of a single node changes. Furthermore, the time interval for the event occurrence is exponentially distributed. This time interval can equivalently be described as the minimum of transition times of a set of statistically independent processes on node states, denoted by $X_i$, and pair states, denoted by $(X_i,X_j)$, as the following:

\begin{align*}
X_i&: I\rightarrow S \mathrm{, \; for } \; i\in V,& T \sim exp(\delta),\\
(X_{i},X_{j})&:(S,I)\rightarrow (I,I)\mathrm{, if }(i,j)\in E,&T\sim exp(\beta w_{ij}),
\end{align*}

where $\delta$ and $\beta$ are called *curing* and *infection* rates, respectively, and $T$ represents the corresponding exponentially distributed transition duration.

Describing the network Markov process as competition among statistically independent nodal and edge-based transitions, similar to the above formulation of the SIS process, allows for a much more general framework for modeling networked epidemic processes (see, [68]). We will use this approach to describe our adaptive contact epidemic model.

Finally, the Kolmogorov equation, which governs probability distribution of the SIS Markov process, is a system of $2^{N}$ coupled differential equations which is neither computationally nor analytically tractable for large number of nodes. Moment closure approximations [39], [68], [69], [70] or Monte Carlo simulations are thus necessary to study the networked SIS process. The SIS process shows a phase transition behavior where initial infections die out quickly for small values of $\beta /\delta$, while infections can persist in the network for long time (coined as *metastable state*) for large values of $\beta /\delta$ [71]. The critical value separating these regions is called the *epidemic threshold*. As such, epidemic threshold suggests a measure of networks robustness against epidemic spreading. In this paper, whenever we say network $a$ is more robust against epidemic spreading than network $b$, we mean network $a$ has a larger value of epidemic threshold that network $b$.

### 4.2 AC-SAIS Markov Model

Consider a population of $N$ individuals, where each individual is either *susceptible*, *alert*, or *infected*. For each individual $i\in \lbrace 1,\ldots, N\rbrace$, let the random variable $X_{i}(t)=S$ if the individual $i$ is susceptible at time $t$, $X_{i}(t)=A$ if alert, and $X_{i}(t)=I$ if infected. In the AC-SAIS model of this paper, contacts of a node depends on her state. Specifically, we define $\mathcal{N}_{i}^{S}$ as the neighbors of node i when she is susceptible, and $\mathcal{N}_{i}^{A}$ as her neighbors when she is alert. Associated with these neighborhood sets, we consider weight values $w^S_{ij}>0$ if $j\in \mathcal{N}_{i}^{S}$ and $w^A_{ij}>0$ if $j\in \mathcal{N}_{i}^{A}$ as a proxy for heterogeneity of the contact levels.

**Fig. 2.**
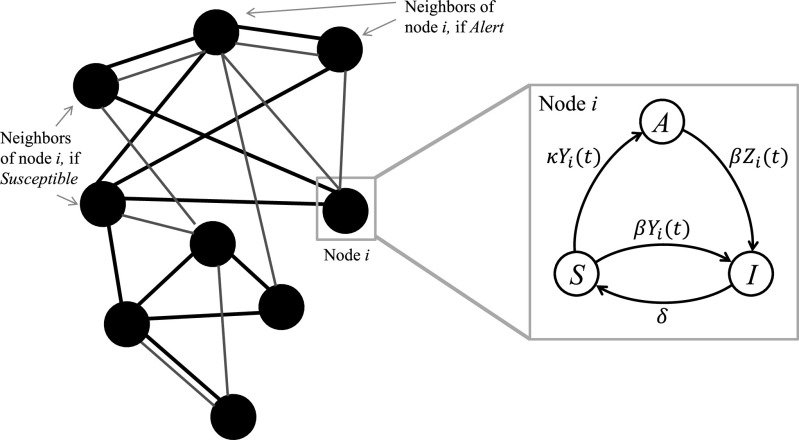
Schematic of the AC-SAIS model. Black edges correspond to neighborhood $\mathcal{N}_{i}^{S}$ of susceptible node $i$, while red edges represent the neighborhood $\mathcal{N}_{i}^{A}$ when node $i$ is in the alert state. Here, $\beta$, $\delta$, and $\kappa$ are the infection rate, curing rate, and alerting rate, respectively. $Y_{i}(t)$ is the number of infected neighbors of $i$ in $\mathcal{N}_{i}^{S}$ at time $t$ and $Z_{i}(t)$ is the number of infected neighbors of $i$ in $\mathcal{N}_{i}^{A}$ at time $t$.
2) *Alerting of susceptible nodes:* A susceptible individual becomes alert from her infected neighbor (among $\mathcal{N}_i^S$) after an exponentially distributed random time duration with the *alerting* rate $\kappa$
  \begin{equation*}
(X_{i},X_{j}):(S,I)\rightarrow (A,I)\mathrm{, \; if } \; (i,j)\in E_S,\quad T\sim exp(\kappa w^S_{ij}).
\end{equation*}
3) *Infection of alert nodes:* An alert individual becomes infected due to having an infected neighbor among her switched neighborhood set $\mathcal{N}_i^A$ after an exponentially distributed random time duration with the infection rate $\beta$
  \begin{equation*}
(X_{i},X_{j}):(A,I)\rightarrow (I,I)\mathrm{, \; if } \; (i,j)\in E_A,\quad T\sim exp(\beta w^A_{ij}).
\end{equation*}
4) *Recovering of infected nodes:* An infected individual recovers to the susceptible state after an exponentially distributed random time duration with recovery rate $\delta$
  \begin{equation*}
X_i: I\rightarrow S \mathrm{, \; for \; }i\in V,\qquad T\sim exp(\delta).
\end{equation*}

Four competing stochastic transitions describe the AC-SAIS model, as Fig. 2 depicts:

1) *Infection of susceptible nodes:* A susceptible individual becomes infected from her infected neighbor (among $\mathcal{N}_i^S$) after an exponentially distributed random time duration with the infection rate $\beta$
  \begin{equation*}
(X_{i},X_{j}):(S,I)\rightarrow (I,I)\mathrm{, \; if } \; (i,j)\in E_S,\quad T\sim exp(\beta w^S_{ij}).
\end{equation*}

*A Few Remarks on the AC-SAIS Model.* The disease dynamics component of the AC-SAIS model is according to the networked SIS model, elaborated in Section 4.1. Therefore, a representative example would be the spread of Syphilis or Gonorrhea for which the sexual contact network is well-defined and disease dynamics are SIS-type. In this scenario, the alerting process can be the result of a partner notification effort.

In the AC-SAIS model, we assume that if an alert individual never gets infected, she will remain in the alert state indefinitely. In other words, we do not consider an awareness decay process where alert individuals can transition to the susceptible state directly. In practice, we are assuming that the awareness decay process is so much slower than the disease dynamics that it becomes irrelevant for the disease spreading. Interested readers can refer to [44] for analysis of an SAIS model with awareness decay.

The current setup of the AC-SAIS model only considers a type-2 preventive behavior of altering contacts, whereas the original SAIS model considered a type-1 preventive behavior by assuming a lower infection rate for alert individuals. It would be possible to also incorporate type-1 behaviors in the AC-SAIS model by lowering the infection rate for the alert individuals to $\beta _a<\beta$. In order to isolate the role of network adaptation, we do not change the infection rate in this study.

The contact alteration scheme in the AC-SAIS model assumes the contacts set of an individual only depends on her own state; it is the default set when susceptible, and the adapted set when alert. Particularly, the contact set of an alert individual is fixed and is independent of the health state of those contacts. Such contact adaptation scheme is most sensible when the identity of infected contacts are not known to the individual. For example, in the context of sexually transmitted diseases and partner notification, the identity of the infectious patient is not revealed to their partners. So, a subsequent contact adaptation may not necessarily lead to definite avoidance of infectious partners.

### 4.3 An Equivalent Multilayer Representation

From a networked dynamical system perspective, the network topology in the AC-SAIS model is time-varying and switches among $2^{N}$ different possibilities because each node $i$ may adopt one of the two neighborhood sets $\mathcal{N}_i^S$ and $\mathcal{N}_i^A$. However, the AC-SAIS model can be equivalently interpreted as a spreading process on a two-layer network. The AC-SAIS Markov process described in Section 4.2 falls in the broad class of generalized epidemic modeling framework (GEMF) introduced in [68] for spreading processes on multilayer networks. In essence, the switching contact network of the AC-SAIS model can be equivalently described as a spreading process on multilayer network $\mathcal{G}=(V,E_{S},E_{A})$, where each layer determines the interaction neighborhood that induces state change in a node, depending on its current state. Note that we only need to include layers for susceptible and alert nodes, because the transition of an infected node towards the susceptible state is spontaneous and does not depend on other nodes states.

Significantly, a multilayer network formulation of adaptive contact reduces the problem from defining a process between $2^{N}$ separate topologies to defining a process on top of a static two–layer network, effectively modeling complex switching dynamics with a conceptually straightforward framework. The network layers $G_{S}=(V,E_S)$ and $G_{A}=(V,E_A)$ represent the two extreme cases among all possible $2^{N}$ configurations. The network layer $G_S$ would be physical contact network if none of the nodes were alert, and the network layer $G_A$ would be the physical contact network if none of the nodes were susceptible. Associated with network layers $G_S$ and $G_A$, we define the weighted adjacency matrices $W_{S}=[w_{ij}^S]$ and $W_{A}=[w_{ij}^A]$, respectively. The realized topology at a given time will be a mixture of the two network layers according to the collective node states at that time. Interestingly though, we show it is possible to characterize the behavior of the AC-SAIS model in terms of the spectral properties of $W_{S}$ and $W_{A}$ and their interrelation.

*Remark.* The actual, physical/social contact between the network agents is fundamentally different from those represented by the multilayer network $\mathcal{G}$. For example, a directed edge $(i,j)\in E_S$ is physically relevant only if node $i$ is susceptible and node $j$ is infected. Otherwise, node $i$ and $j$ might have a different interaction if, for instance, both are susceptible. However, the later is not relevant for disease spreading and thus no need to be incorporated in our epidemic model. Take for instance the contact between node $i$, who is a nurse, and node $j$, who is a student. These two might not have any social contact in normal situation, however, when node $j$ (the student) is sick, she can possibly pass infection to node $i$ (the nurse); and this is the contact important for epidemic modeling purpose. Also, realize that this contact is directional because when the nurse is sick, she may not have a physical contact with the student. This is why in our state-dependent contact network formulation we do not make “undirectedness” assumption on the underlying graph.

### 4.4 Mean-Field AC-SAIS Model

Similar to the networked SIS model described in Section 4.1, the collective state $\boldsymbol{X}(t)$ in the AC-SAIS model is a Markov process. However, this Markov process is both analytically and numerically intractable due to its exponential state space size of $3^{N}$ (each node can be in one of three states). We can leverage the observation that the AC-SAIS model falls in the GEMF class of stochastic spreading processes on multilayer networks—for which Sahneh et al. [68] have derived a system of nonlinear differential equations describing the evolution of state-occupancy probabilities after adopting a first-order, mean-field-type approximation.

Following procedures explained in [68], we find the first order mean-field-type approximate model for the AC-SAIS model as

\begin{equation*} \dot{p}_{i} = -\delta p_{i}+\beta (1-q_{i}-p_{i})\sum w_{ij}^{S}p_{j}+\beta q_{i}\sum w_{ij}^{A}p_{j}, \end{equation*}

\begin{equation*} \dot{q}_{i} = \kappa (1-q_{i}-p_{i})\sum w_{ij}^{S}p_{j}-\beta q_{i}\sum w_{ij}^{A}p_{j}, \end{equation*}

for $i\in \lbrace 1,\ldots, N\rbrace$, where $p_{i}$ corresponds to the probability that individual $i$ is infected, and $q_{i}$ corresponds to the probability that she is alert.

It is worthwhile to acknowledge the limitations of mean-field models. Statistical physics tells us that MF approximations function suitably for infinite-dimensional networks. While, they can perform very poorly for sparse or highly structured networks, such as rings or low-dimensional lattices, particularly close to critical model parameters. Despite, the approximation allows for investigating extremely complex dynamics, and discovering intriguing phenomena and key network characteristics influencing them.

## 5 Analysis of AC-SAIS Model

In this section, we compute and study the epidemic threshold of the mean-field AC-SAIS model in Eqs. ((2) and (3)) through analyzing its equilibrium points. Our motivation for this approach stems from the mean-field SIS model which exhibits a threshold phenomena in its equilibrium where a stable (see, [72], [73], [74]) endemic equilibrium emerges [39].

To facilitate the subsequent analysis, we make the following assumption on the structure of the default and adapted neighborhoods throughout this article.

**Assumption 1.**

The edge sets $E_{S}$ and $E_{A}$ are such that the two-layer network $\mathcal{G}=(V,E_{S},E_{A})$ is M–connected according to Definition 5.

### 5.1 Mean-Field Epidemic Threshold Equation

Our approach to finding the critical value $\tau _c$ for AC-SAIS model (Eqs. (2) and (3)) is through examining the equilibrium points; as used by Van Mieghem for the SIS model in [39]. The idea is to show that for $\tau >\tau _c$ an endemic equilibrium ($p^*_i>0,\ 
\forall i)$ exists aside from the disease-free equilibrium.^8^8.Note that $\tau _c$ is the mean-field model threshold value which is a lower bound of the actual value in the exact stochastic SIS process. In this approach, strong connectivity of the underlying contact network is pivotal. In case of the SIS model, Van Mieghem [39] showed that if the contact graph is strongly connected, equilibriums of the mean-field model must either be all zero—the disease-free equilibrium—or they must be strictly positive—the endemic equilibrium. Following lemma shows that similar argument holds for the AC-SAIS model (Eqs. (2) and (3)) under the M–connectivity assumption of the multilayer network $\mathcal{G}$ as in Definition 5.

**Lemma 4.**

Under Assumption 1, the equilibrium value of the infection probability $p_{i}^{\ast }$ is either zero for all individuals, or strictly positive for all individuals. Moreover, a positive equilibrium satisfies

\begin{equation*} \frac{p_{i}^{\ast }}{1-p_{i}^{\ast }}=\tau \left\lbrace \frac{(\bar{\kappa }+1)\sum w_{ij}^{A}p_{j}^{\ast }\sum w_{ij}^{S}p_{j}^{\ast }}{\bar{\kappa }\sum w_{ij}^{S}p_{j}^{\ast }+\sum w_{ij}^{A}p_{j}^{\ast }}\right\rbrace, \end{equation*}

with *e*ffective infection rate $\tau$ and *r*elative alerting rate $\bar{\kappa }$ respectively defined as^9^9.According to Poisson processes theory, the effective infection rate $\tau =\beta /\delta$ is equal to the expected number of attempts per link that an infected node makes to infect her neighbor during her infectious period [75]. The relative alerting rate $\bar{\kappa }=\kappa /\beta$ indicates the ratio of the chance that an infected neighbor cause her neighbor to become alert versus the causing her to become infected. For instance, $\bar{\kappa }=\frac{1}{2}$ means the chance that a node becomes infected from her infected neighbor is twice the chance of becoming alert as the result of interacting with the same neighbor.

\begin{equation*}
\tau \triangleq \beta /\delta, \quad \bar{\kappa }\triangleq \frac{\kappa }{\beta }.
\end{equation*}

**Proof.**

Assume $p_{j}^{\ast }>0$. Letting $\dot{q}_i=0$ in Eq. (3) for any node $i$ with $w_{ij}^{S}>0$ or $w_{ij}^{A}>0$, yields

\begin{equation*} q_{i}^{\ast }=\frac{\bar{\kappa }\sum w_{ij}^{S}p_{j}^{\ast }}{\bar{\kappa }\sum w_{ij}^{S}p_{j}^{\ast }+\sum w_{ij}^{A}p_{j}^{\ast }}(1-p_{i}^{\ast }). \end{equation*}

Therefore, according to Eq. (2), the equilibrium infection probabilities $p_{i}^{\ast }$ satisfy

\begin{equation*} p_{i}^{\ast }=\beta \frac{(1-q_{i}^{\ast })\sum w_{ij}^{S}p_{j}^{\ast } +q_{i}^{\ast }\sum w_{ij}^{A}p_{j}^{\ast }}{\delta +\beta \sum w_{ij}^{S} p_{j}^{\ast }}. \end{equation*}

Replacing $q^*_i$ from Eq. (5) in Eq. (6) yields the formula in Eq. (4).

The rest of the proof concerns choosing $i$ deliberately, so that $p_{j}^{\ast }>0$ guarantees $p_{i}^{\ast }>0$, and repeating the process until concluding positive equilibrium probabilities for all nodes. We employ the definition of graphs $\boldsymbol{G}^k=(\mathcal{P}_k,\mathcal{L}_k)$ associated with the multilayer network $\mathcal{G}=(V,E_S,E_A)$ as explained in Section 3.2. According to Definition 5, if $\mathcal{G}$ is M–connected, there exists $k_*$ such that $\boldsymbol{G}^{k_*}$ is a strongly connected graph. Eq. (4) indicates that in order to get $p^*_i>0$, both $\sum w_{ij}^{A}p_{j}^{\ast }$ and $\sum w_{ij}^{S}p_{j}^{\ast }$ must be positive. Therefore, choosing $i$ such that $(i,j)\in \mathcal{L}_1$ (for which $w_{ij}^S>0$ and $w_{ij}^A>0$) necessitates $p_{i}^{\ast }>0$. Repeating this process yields the equilibrium probability of all the nodes in the strongly connected component of $G^{1}$ that contains $j$ are all positive. This strongly connected component of $G^{1}$ becomes a single hypernode, which we call $J\in \mathcal{P}_2$, for graph $\boldsymbol{G}^2$. So far, we have proved that $\forall j\in J,\ 
p^*_j>0$. According to the definition of $\boldsymbol{G}^k$, for graph $\boldsymbol{G}^2$, there is a directed link from component $J$ to component $I$, i.e., $(I,J)\in \mathcal{L}_2$, if and only if

\begin{equation*} \exists i\in I \;\; \mathrm{such \; that} \;\;w^{A}_{ij_{1}}, w^{S}_{ij_{2}}>0 \;\;\mathrm{for \; some} \;\; j_{1},j_{2}\in J. \end{equation*}

Since $\forall j\in J,\ 
p^*_j>0$, we get $p^*_i>0$ for the above choice of $i$, which further indicates all the nodes of $I$ have positive equilibrium values. As a result, all the nodes belonging to the strongly connected component of $\boldsymbol{G}^2$ that contains $J$ have positive equilibrium values. This procedure can be repeated for $\boldsymbol{G}^3,\ldots, \boldsymbol{G}^{k_*}$. Since, $\boldsymbol{G}^{k_*}$ is strongly connected, all the nodes of the network must have positive equilibrium values.

We can find the epidemic threshold by examining the equilibrium points in Eq. (4). For $\tau <\tau _{c}$, the disease-free state is the only equilibrium. However, for $\tau >\tau _{c}$, another equilibrium point $\boldsymbol{p}^{\ast }\triangleq [p_{1}^{\ast },\ldots, p_{N}^{\ast }]^{T}\succ 0$, also exists in the positive orthant. Therefore, we find the threshold value of $\tau _{c}$ if we can find a critical value $\tau =\tau _c$ such that $p_{i}^{\ast }|_{\tau =\tau _{c}}=0$ while $\frac{dp_{i}^{\ast }}{d\tau }|_{\tau =\tau _{c}}>0$ for all $i\in \lbrace 1,\ldots, N\rbrace$. We have the following theorem regarding the value of the epidemic threshold. We would like to emphasize that such a threshold corresponds to the mean-field approximate model (Eqs. (2) and (3)) and should not be confused as the actual threshold value in the exact AC-SAIS Markov model.

**Theorem 6.**

The threshold value $\tau _{c}$ for AC-SAIS model ((2) and (3)) is such that the equation

\begin{equation*} \boldsymbol{z}=\tau _{c}(\bar{\kappa }+1)F(\boldsymbol{z}), \end{equation*}

with

\begin{equation*} F(\boldsymbol{z})_{i}\triangleq \frac{\sum w_{ij}^{A}z_{j}\sum w_{ij}^{S}z_{j}}{\bar{\kappa }\sum w_{ij}^{S}z_{j}+\sum w_{ij}^{A}z_{j}}, \end{equation*}

has a nontrivial solution $\boldsymbol{z}\triangleq [z_{1},\ldots, z_{N} ]^{T}\succ 0$.

**Proof.**

Eq. (4) can be rewritten as

\begin{equation*}
\frac{p_{i}^{\ast }}{\sum w_{ij}^{S}p_{j}^{\ast }}=\tau ({1-p_{i}^{\ast }})\Bigg\lbrace \frac{(\bar{\kappa }+1)\sum w_{ij}^{A}p_{j}^{\ast }}{\bar{\kappa }\sum w_{ij}^{S}p_{j}^{\ast }+\sum w_{ij}^{A}p_{j}^{\ast }}\Bigg\rbrace .
\end{equation*}

Now, we take the limit of both sides as $\tau \downarrow \tau _c$, for which $p_i^*\downarrow 0$ for $\forall i$ according to the definition of an epidemic threshold. Since the limit of numerator and denominator of fraction terms of both sides goes to zero, we apply the L’Hôpital’s rule for limits [76]

\begin{equation*}
\lim \limits _{\tau \downarrow \tau _c}\frac{ \frac{d}{d\tau }p_{i}^{\ast }}{{\sum w_{ij}^{S}\frac{d}{d\tau }p_{j}^{\ast }}}=\tau _c\lim \limits _{\tau \downarrow \tau _c}\frac{(\bar{\kappa }+1)\sum w_{ij}^{A}\frac{d}{d\tau }p_{j}^{\ast }}{\bar{\kappa }\sum w_{ij}^{S}\frac{d}{d\tau }p_{j}^{\ast }+\sum w_{ij}^{A}\frac{d}{d\tau }p_{j}^{\ast }}.
\end{equation*}

Defining $z_{i}\triangleq \frac{d}{d\tau }p_{i}^{\ast }|_{\tau =\tau _{c}}$, the above equation will lead to (8). The value of $\tau _{c}$ that solves (8) is the critical value for which $p_{i}^{\ast }=0$, however, $dp_{i}^{\ast }/d\tau >0$, denoting a second-order phase transition at $\tau =\tau _{c}$. Therefore, $\tau _{c}$ is the epidemic threshold for AC-SAIS model ((2) and (3)).

Letting $\bar{\kappa }=0$ in Eq. (9) yields $F(\boldsymbol{z})=W_S\boldsymbol{z}$, which reduces Eq. (8) to the Perron Frobenius problem $\boldsymbol{z}=\tau _{c}W_{S}\boldsymbol{z}$, suggesting $\tau _{c}=1/\lambda _{1}(W_{S})$; the SIS mean-field threshold. For the AC-SAIS model, the epidemic threshold condition pertains to the nonlinear Perron-Frobenius problem (8). Though an analytical solution is not expected, we can employ the tools of Section 3.1.

In order to employ Theorem 6, we should prove our nonlinear map $F$ in Eq. (9) is homogeneous and concave, and it satisfies condition **C2** defined in Definition 3. The map $F$ in Eq. (9) is defined for interior of the nonnegative cone. We extend the definition to the boundary of the nonnegative cone by letting $F(\boldsymbol{z})_i=0$ whenever $\sum w_{ij}^{S}z_{j}=0$ and $\sum w_{ij}^{A}z_{j}=0$. In this way, $F(\boldsymbol{z})$ is well defined for all $\boldsymbol{z}\succeq 0$. It is obvious that $F$ in Eq. (9) is a homogeneous map. Concavity of $F$ can be also deduced from the concavity of the function $g:\mathbb {R}^{2}_{+}\rightarrow \mathbb {R}_{+}$ defined as $g(u,v)=\frac{uv}{u+v}$ (which is half of the harmonic average) because the arguments of $u$ and $v$ are linear transformation of $z_i$’s. Next lemma proves that it also satisfies condition **C2**.

**Lemma 5.**

Function $F$, defined in Eq. (9), satisfies condition **C2** if and only if the multilayer graph $\mathcal{G}=(V,E_S,E_A)$ is M–connected.

**Proof.**

We just argued that $F$, as defined in Eq. (9), is homogeneous and concave. Also, for any set $J$, $F_i(e_J)>0$ if and only if there exists $i\notin J$ and $j_1,j_2\in J$ for which $w_{i,j_1}^S>0$ and $w_{i,j_2}^A>0$, i.e., $(i,j_1)\in E_S$ and $(i,j_2)\in E_A$. Therefore, Theorem 5 is applicable and proves the lemma.

Since we showed $F$ in Eq. (9) is homogeneous and concave, and satisfies condition **C2**, we can apply Theorem 4 to prove existence and uniqueness of a strictly positive solution for $\boldsymbol{z}$ to the nonlinear Perron–Frobenius problem (8).

**Corollary 1.**

If the multilayer graph $\mathcal{G}=(V,E_S,E_A)$ is M–connected, the nonlinear Perron–Frobenius problem (8) has a unique solution $\boldsymbol{z}=\boldsymbol{z}_*\succ 0$ with $||\boldsymbol{z}_*||_2=1$. Furthermore, the following numerical update law will converge asymptotically to $\boldsymbol{z}_*$

\begin{equation*} \boldsymbol{z}_{k+1}\triangleq \frac{F(\boldsymbol{z}_{k})+c\boldsymbol{z}_{k}}{\left\Vert F(\boldsymbol{z}_{k})+c\boldsymbol{z}_{k}\right\Vert _{2}}, \end{equation*}

with $c>0$, and the initial state $\boldsymbol{z}_{0}\succ 0$ and $||\boldsymbol{z}_{0}||_2=1$. Moreover, the threshold value is $\tau _{c}=\frac{1}{(\bar{\kappa }+1)(\boldsymbol{z}_*^{T}F(\boldsymbol{z}_*)-c)}$.

### 5.2 Possible Solutions to MF Epidemic Threshold

**Fig. 3.**
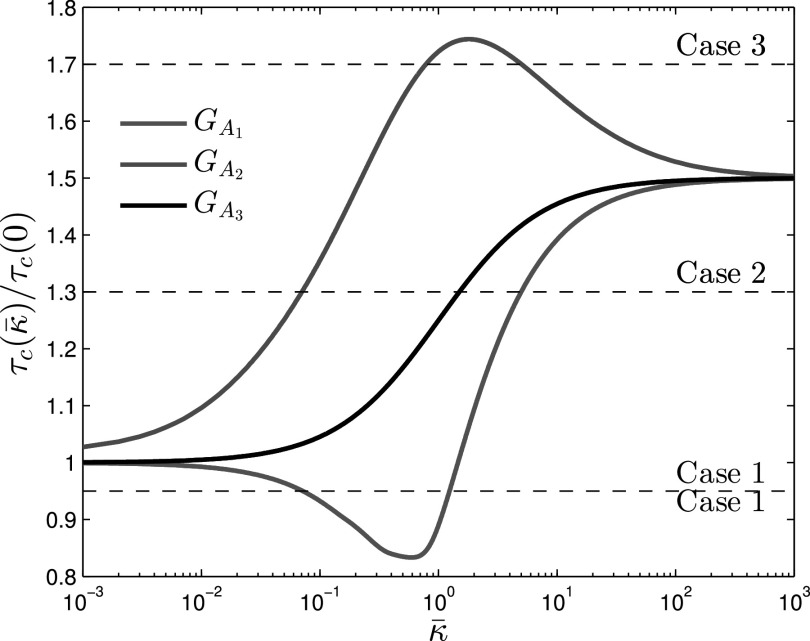
Normalized epidemic threshold $\tau _{c}(\bar{\kappa })/\tau _c(0)$ as a function of relative alerting rate $\bar{\kappa }=\frac{\kappa }{\beta }$, showing three dependency scenarios. All three alert layers have the same spectral radius with respect to $G_{S}$ i.e., $\lambda _{1}(W_{S})/\lambda _{1}(W_{Ai})=1.5$. Therefore, in all of them the threshold value $\tau _c(\bar{\kappa })$ starts from $\tau _c(0)=1/\lambda _1(W_S)$ and converges to $\tau _c(\infty)=1.5\tau _c(0)$. Graph $G_{A1}$ is synthesized such that $\Psi (W_S,W_{A1})<1$. From the red curve we can observe that $\tau _{c}(\bar{\kappa })$ decreases for small $\bar{\kappa }$ values after which it increases. Graph $G_{A2}$ is synthesized such that $\Psi (W_{A2},W_S)>1$. In this case the blue curve $\tau _{c}(\bar{\kappa })$ is maximal around $\bar{\kappa }\approx 2$. The topology of graph $G_{A3}$ is $G_{S}$ with reduced weights and is represented by the black epidemic threshold curve which increases monotonically by $\bar{\kappa }$.

Corollary 1 proves the existence and uniqueness of a solution for the AC-SAIS threshold formula in Eq. (8). Furthermore, the update law of Eq. (10) suggests a numerical algorithm for finding the threshold value. Interestingly, a numerical experiment in the next section (see, Fig. 3) shows that the epidemic threshold value is a non-monotone function of contact adaptation rate (quantified by $\bar{\kappa }$); indicating faster contact adaptation is not necessarily always better in suppressing epidemics. Here, we aim to predict such scenarios without numerically solving the nonlinear Perron-Frobenius problem for the epidemic threshold.

The idea is perturbing the threshold Eq. (8) around two extreme cases of $\bar{\kappa }=0$ and $\bar{\kappa }\rightarrow \infty$, for which we know the exact solutions. Specifically, 1) for $\bar{\kappa }=0$, the epidemic threshold is $\tau _{c}|_{\bar{\kappa }=0}=\frac{1}{\lambda _{1}(W_{S})}$ and $\boldsymbol{z}|_{\bar{\kappa }=0}=\boldsymbol{v}_{S}$ is a solution, where $\boldsymbol{v}_{S}$ is the dominant eigenvector of matrix $W_{S}$; and 2) for $\bar{\kappa }\rightarrow \infty$, the epidemic threshold is $\tau _{c}|_{\bar{\kappa }\rightarrow \infty }=\frac{1}{\lambda _{1}(W_{A})}$ and $\boldsymbol{z}|_{\bar{\kappa }\rightarrow \infty }=\boldsymbol{v}_{A}$ is a solution, where $\boldsymbol{v}_{A}$ is the dominant eigenvector of matrix $W_{A}$. Thus, employing spectral perturbation techniques, we can approximate the threshold value for small and large values of relative alerting rate $\bar{\kappa }$.

**Theorem 7.**

The value of the epidemic threshold solving (8) has the forms

\begin{equation*} \tau _{c}(\bar{\kappa }) =\frac{1}{\lambda _{1}(W_{S})}(1+\bar{\kappa }(\Psi (W_{S}, W_{A})-1))+o(\bar{\kappa }), \end{equation*}

suitable for small values of $\bar{\kappa }$, and

\begin{equation*} \tau _{c}(\bar{\kappa })=\frac{1}{\lambda _{1}(W_{A})}(1+\bar{\kappa }^{-1}(\Psi (W_{A}, W_{S})-1))+o(\bar{\kappa }^{-1}), \end{equation*}

suitable for large values of $\bar{\kappa }$, where $\Psi (A,B)$ is

\begin{equation*} \Psi (A,B)\triangleq \sum \nolimits _{i=1}^{N}u_{i}v_{i}\frac{\sum \nolimits _{j=1} ^{N}a_{ij}v_{j}}{\sum \nolimits _{j=1}^{N}b_{ij}v_{j}}, \end{equation*}

and $\boldsymbol{v}_A=[v_{1},\ldots, v_{N}]^{T}$ and $\boldsymbol{u}_A=[u_{1}, \ldots, u_{N}]^{T}$ are the right and left dominant eigenvectors of $A$ corresponding to $\lambda _{1}(A)$ with $\boldsymbol{v}_A^T\boldsymbol{u}_A=1$.

Fortunately, spectral perturbation of the nonlinear Perron-Frobenius problem (Eq. (8)) leads to analytically tractable formulas expressed in terms of spectral properties of individual layers $G_S$ and $G_A$, and their interrelation (as manifested by $\Psi$ terms in Eqs. (11) and (12)). Using Eq. (11) for small values of $\bar{\kappa }$ and Eq. (12) for large values of $\bar{\kappa }$, we can categorize several solution possibilities for the full range of $\bar{\kappa }$ values.

To reflect more realistic scenarios, we impose the constraint $\lambda _{1}(W_{S})>\lambda _{1}(W_{A})$, that is, we assume that if all healthy individuals adopted their alert neighborhood simultaneously, they would collectively raise the epidemic threshold value, making their network more robust against epidemics than the default contact graph $G_{S}$.

The three scenarios for the dependency of the threshold value on contact adaptation rate—as shown in Fig. 3—can be characterize as the following:

1) *Monotone scenario* (the faster, the better): This is the simplest case where the value of the epidemic threshold increases monotonically with $\bar{\kappa }$, as simulated in Section 6 and shown in Fig. 3 by the black curve. The monotone behavior happens if $\Psi (W_{S},W_{A})>1$ and $\Psi (W_{A},W_{S})<1$. Such monotonically increasing curve occurs, for instance, in contact-avoidance cases^10^10.This is because having $w_{ij}^A\leq w_{ij}^S$ yields $\sum \nolimits _{j=1}^{N}w_{ij}^{S}v_{Sj}>\sum \nolimits _{j=1}^{N} w_{ij}^{A}v_{Sj}$ and $\sum \nolimits _{j=1}^{N}w_{ij}^{S}v_{Aj}>\sum \nolimits _{j=1}^{N}w_{ij}^{A}v_{Aj}$, which according to Eq. (13), leads to $\Psi (W_{S},W_{A})>1$ and $\Psi (W_{A},W_{S})<1$. where $w_{ij}^A\leq w_{ij}^S$. In other words, if individuals only reduce contact with their neighbors upon becoming alert, the higher the rate they do so, the better; because the epidemic threshold increases with the alerting rate in this scenario.
2) *Overshooting scenario* (moderate even better than fast): It is possible that an optimal alerting rate $\bar{\kappa }$ exists for which the adaptive network is most robust with respect to spreading infection. In other words, having a moderate contact adaptation rate is even better than than the case where the alerting rate is so large that alerting processes is almost instantaneous. The blue curve in Fig. 3 corresponds to this case. This scenario happens if $\Psi (W_{A},W_{S})>1$.
3) *Undershooting scenario* (adaptation goes wrong if slow): An interesting and important scenario is when $\Psi (W_{S},W_{A})<1$. In this case, the value of the epidemic threshold initially decreases as the value of $\bar{\kappa }$ increases. If the switching rate is not fast enough, the alerting process can unintendedly worsen the infection spreading compared to keeping the default contacts! The red curve in Fig. 3 depicts such scenario.

The following lemma shows that asymmetry of contacts is critical for observing the latter scenario.

**Lemma 6.**

If $W_{S}$ and $W_{A}$ are both symmetric, $\Psi (W_{S},W_{A})$ is lower-bounded as

\begin{equation*} \Psi (W_{S},W_{A})\geq \frac{\lambda _{1}(W_{S})}{\lambda _{1}(W_{A})}. \end{equation*}

Given $\lambda _{1}(W_{S})>\lambda _{1}(W_{A})$ (the alert layer is more robust than the susceptible layer), the right hand side of Eq. (14) will be always greater than 1. Hence, for undirected network layers, it is impossible for the critical threshold of the adaptive contact network to go below the critical threshold of the default contacts layer, $G_{S}$. We can conclude that asymmetry of contacts is in part responsible for this unexpected behavior.

## 6 Numerical Experiments

In this section, we perform a numerical study to evaluate our findings. For $E_{S}$ edges, we consider the well-known “Football” network from [77] with $N=115$ nodes and $|E_S|=615$ edges, and spectral radius $\lambda _{1}(W_{S})=10.8$. Given $G_S$, we synthesize three adapted contact layers $G_{A1}$, $G_{A2}$, and $G_{A3}$ as described bellow, and compute their corresponding threshold values as a function of the relative alerting rate as shown in Fig. 3.

• The spectral radii of $G_{Ai}$ graphs are all equal to $\frac{2}{3}$ of the spectral radius of $G_{S}$, i.e., $\lambda _{1}(W_{Ai}) = \frac{2}{3} \lambda _{1}(W_{S})$. In this way, we ensure that the adapted contacts layers are more robust to epidemic spreading compared to the default contacts layer. This can be verified in Fig. 3 where $\tau _c(\infty)=\frac{3}{2}\tau _c(0)$. Note that $\tau _c(0)$ is the threshold value when $\bar{\kappa }=0$, i.e., no adaptation occurs, and $\tau _c(\infty)$ corresponds to $\bar{\kappa }=\infty$ where the contact adaptation occurs instantaneously.
• For $G_{A1}$, $\Psi (W_{S},W_{A1}) < 1$. From Eq. (11), we can predict that for small values of $\bar{\kappa }$, the epidemic threshold decreases below $\tau _c(0)$, the threshold if no contact adaptation was in place at all . Therefore, we expect an undershoot in $\tau _{c}(\bar{\kappa })$ as a function of $\bar{\kappa }$. This is the configuration where contact adaptation can “go wrong”; despite the fact that the alert contact network is more robust, switching to it can adversely aid in the spread of infection. The red curve in Fig. 3 corresponds to this scenario.
• For $G_{A2}$, $\Psi (W_{A2},W_{S})>1$. From Eq. (12), we can predict it is possible to get $\tau _c(\bar{\kappa })>\tau _c(\infty)$, an thus there is a value for $\bar{\kappa }$ for which $\tau _{c}(\bar{\kappa })$ is maximum. This is in contrast to $G_{A1}$ in that the epidemic threshold for the multilayer network is greater than its constituent layers. In this configuration, the characteristics are such that an enhanced robustness is created synergistically. The blue curve in Fig. 3 corresponds to this scenario.
• Graph $G_{A3}$ is made by decreasing the link weights from $G_{S}$, representing a social-avoidance scenario. As discussed in Section 5.2, we expect to see a monotonic increase in the epidemic threshold as the contact adaptation rate increases. The black curve in Fig. 3 corresponds to this scenario.

**Fig. 4.**
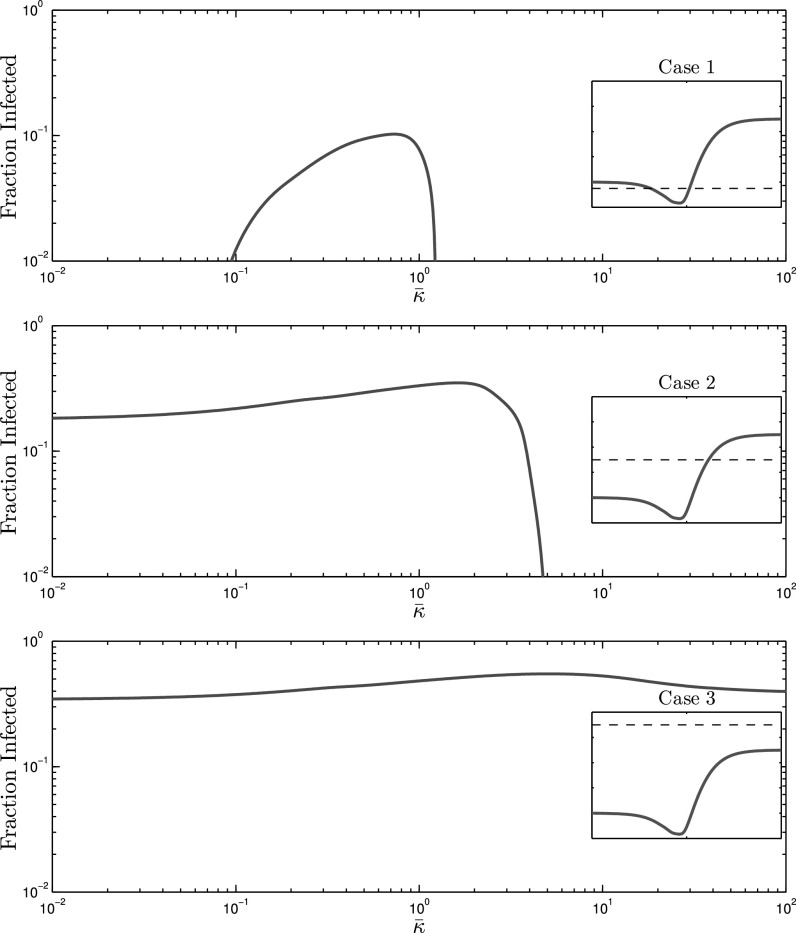
The effect of alerting rate on infection size for the undershooting scenario, for which the epidemic threshold dependence on $\bar{\kappa }$ is depicted by the red curve in Fig. 3. *Case 1* (top) Despite setting the effective infection rate below that of the extreme cases, i.e., $\tau <\tau _c(0)<\tau _c(\infty)$, an epidemic outbreak is still observed for small alerting rates because $\tau$ is larger than the minimum of $\tau _c(\bar{\kappa })$. *case 2* (middle) Effective infection rate lies in between the two extreme values, i.e., $\tau _c(0)<\tau <\tau _c(\infty)$. There is a slight increase in infected individuals after which the infection size drops to 0 due to the increase in the critical threshold. *Case 3* (bottom) Persistent infections are observed regardless of contact adaptation rate because $\tau _c(\bar{\kappa })<\tau$ for all $\bar{\kappa }$.

**Fig. 5.**
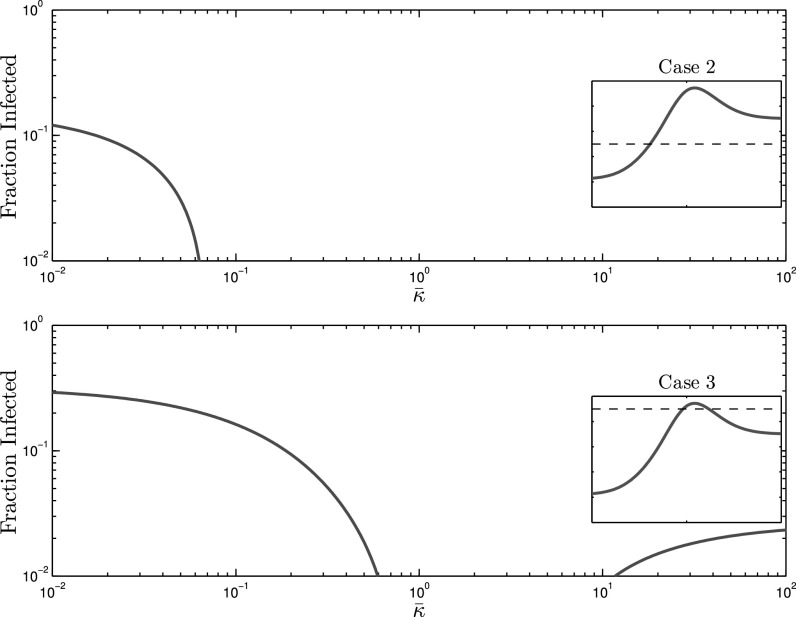
The effect of alerting rate on infection size for the overshooting scenario, for which the epidemic threshold dependence on $\bar{\kappa }$ is depicted by the blue curve in Fig. 3. *Case 1* This case is omitted since the infection size would be 0 regardless of the alerting rate. *Case 2* (top) The behavior is similar to case 2 with $G_{A1}$ (middle graph in Fig. 4) though the transition to zero infection size occurs at a smaller alerting rate. *Case 3* (bottom) This is a scenario when the effective infection rate is larger than the extreme values $(\tau _c(0)<\tau _c(\infty)<\tau)$, yet it is less than the maximum of the threshold curve $\tau _c(\bar{\kappa })$ as seen by the blue curve in Fig. 3. A non-zero infection size is observed for small alerting rates, eventually $\tau _c(\bar{\kappa })$ raises above $\tau$ so that an epidemic cannot be sustained. As the threshold converges towards $\tau _c(\infty)$, an epidemic can once again persist, and the infection size even increases by the contact adaptation rate.

**Fig. 6.**
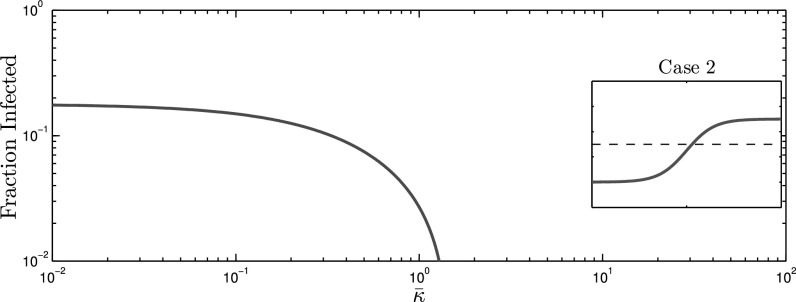
The effect of alerting rate on infection size for the monotone scenario, for which the epidemic threshold dependence on $\bar{\kappa }$ is depicted by the black curve in Fig. 3. *Case 2* Similar to Sections 6.1 and 6.2, case 2 shows a transition between low and high alerting rates where epidemic outbreaks occur for the former and not the latter. Cases 1 and 3 are omitted for trivial behavior.

In order to synthesize $G_{A1}$ and $G_{A2}$, we performed a greedy search to obtain desired values of $\Psi$ functions. For each alert contact graph, $G_{Ai}$, and subsequent multilayer network representation, $\mathcal{G}_i=(V,E_{S},E_{Ai})$, we examine spreading behavior at three effective infection rates $\tau _1=0.9\tau _c(0)$, $\tau _2=1.3\tau _c(0)$, and $\tau _3=1.7\tau _c(0)$, as seen in Fig. 3 (dotted lines). In our numerical simulations, we have set $\delta =1$, which without loss of generality, chooses the unit of time equal to the expected curing period. Steady-state solutions to the mean-field AC-SAIS Eqs. (2) and (3) are calculated for $10^{-2} \leq \bar{\kappa } \leq 10^2$ and fraction of population infected $\bar{p}=\frac{1}{N}\sum _{i=1}^{N}p_i$—as the indicator of severity of the spreading—is plotted as a function of the alerting rate in Figs. 4, 5, and 6.

### 6.1 Adaptation Gone Wrong

For the multilayer network with $G_{A1}$ as the adaptive contact layer, we expect to observe increased epidemic sizes—due to a decreased threshold (red curve of Fig. 3)—for a range of low alerting rates.

In *Case 1*, the infection rate is chosen so that $\tau <\tau _c(0)<\tau _c(\infty)$. In the top plot of Fig. 4, we can see that for most $\bar{\kappa }$ values there is no outbreak, as one would expect since the effective infection rate is below the either extreme values. However, for $0.1 \leq \bar{\kappa } \leq 1.2$ an epidemic is sustained due entirely to inter-layer dynamics creating conditions where an epidemic is more effectively carried throughout the population. In the context of persons altering who they come into contact with, although in an effort to avoid becoming infected, may in fact unintentionally contribute to the opposite outcome.

For *Case 2*, with $\tau _c(0)<\tau <\tau _c(\infty)$, we observe two regimes of behavior as depicted in the middle plot of Fig. 4: for lower alerting rates, where the effective infection rate is above the epidemic threshold $\tau _c(\bar{\kappa })$, an infection is sustained. For higher alerting rates the reverse is true since the critical threshold goes above $\tau$.

In *Case 3*, effective infection rate is set above the critical threshold (red curve of Fig. 3) for all values of $\bar{\kappa }$, i.e., $\tau _c(\bar{\kappa })<\tau$. Therefore, persistent infections are observed regardless of contact adaptation rate in bottom plot of Fig. 4.

### 6.2 Enhanced Robustness

We perform the same computations on when the adapted contact layer is $G_{A_2}$. *Case 1* yields trivially zero infection size. For *case 2*, shown in the top plot of Fig. 5, we observe that increasing alerting rate beyond a certain value successfully suppresses the infection spreading. *Case 3*, shown in the bottom plot of Fig. 5, provides an interesting observation in that the critical threshold raises even larger than the alert contacts layer, indicating that a moderate rate of contact adaptation is indeed better than fast rates in enhancing the robustness of the network. Therefore, for $0.8<\bar{\kappa }<5$, the critical threshold increases such that no infection is sustained. While for larger values an outbreak occurs, and the infection size increases as contact adaptation rate increases.

### 6.3 Monotonic Dependency

In the third scenario, the adapted contact layer is constructed by lowering the edge weights of $G_S$. This would correspond to a social distancing scenario, where individuals limit or abandon their contacts when they become alert. As can be seen by the black curve in Fig. 3, the threshold value increases monotonically by the alerting rate. Fig. 6 depicts the second case where $\tau _c(0)<\tau <\tau _c(\infty)$. As expected, there is a certain value of $\bar{\kappa }*$ so that the epidemic infection is controlled for alerting rates $\bar{\kappa }\geq \bar{\kappa }*$. Case 1 and 3 are omitted for trivial behavior.

## 7 Discussions and Conclusion

The state-dependent switching (adaptive) contact network in the AC-SAIS model leads to rich dynamics for the epidemic spreading process and behavior not yet identified in literature (to the best of the authors’ knowledge). Intuitively, when nodes can “switch” to a neighborhood constituting a more robust network, the expected effect on the overall robustness of the network would be to increase monotonically with the alerting rate. As shown in Sections 6.2 and 6.1, this is not always the case. Indeed, we observed non-monotone dependency of the epidemic threshold in most of our experiment trials. We show how the adaptive switching topology of the contact network is different from fixed static graphs and can lead to regimes of extreme or unexpected behavior. In particular, it is possible that adaptive behavior towards a supposedly more resilient network can in fact worsen the severity of an outbreak, or enable the possibility where it did not exist before. On the other hand, it is possible to configure network layers such that the multilayer network is more robust than either individual layer.

It is noteworthy to mention that some results in the literature point to the observation that contact adaptation do not always aid suppressing the infection. For example, Meloni et al. [26] considered a self-initiated behavior where individuals change their mobility patterns. When travelers decide to avoid locations with high levels of infection and travel through locations with low levels of infections, this behavioral change may facilitate disease spreading because individuals effectively act as vectors of the disease transmission. It is very important to highlight the difference of the underlying mechanism between these formerly reported results and the “adaptation-gone-wrong” behavior in this paper. In our model, individuals who adapt their contacts (alerts) do not act as vectors for propagating the infection because they do not carry infection. This comes purely as a result of the adaptive behavior, signifying the importance of further analysis of state-dependent networks.

Finally, we would like to highlight several aspects of this study that go beyond the specific epidemic model considered in this paper. We developed a necessary and sufficient condition for existence and uniqueness of a positive eigenvector for homogeneous, concave maps. Furthermore, our utilization of multilayer networks to formulate dynamics on state-dependent switching networks is novel and can facilitate analysis of many networked dynamical systems. In our analysis, we come up with concepts that are genuine and novel to multilayer networks. Specifically, our analysis leads to 1) critical phenomena characterized by a nonlinear Perron Frobenius equation, and 2) the definition of M–connectivity. Our proposed concept of M–connectivity can easily scale to more than two layers. Furthermore, the joint descriptor $\Psi$ in Eq. (13) emphasizes the importance of joint descriptors when characterizing dynamics over multilayer networks. While network science has flourished in understanding intra-layer network topologies, intrinsic descriptors of inter-layer connectivity of multilayer networks are yet to be investigated.
